# Depth-Guided Bilateral Grid Feature Fusion Network for Dehazing

**DOI:** 10.3390/s24113589

**Published:** 2024-06-02

**Authors:** Xinyu Li, Zhi Qiao, Gang Wan, Sisi Zhu, Zhongxin Zhao, Xinnan Fan, Pengfei Shi, Jin Wan

**Affiliations:** 1 Hubei Technology Innovation Center for Smart Hydropower, Wuhan 430019, China; 2 College of Information Science and Engineering, Hohai University, Changzhou 213000, China; 3 College of Computer Science and Software Engineering, Hohai University, Nanjing 210098, China; 4 College of Artificial Intelligence and Automation, Hohai University, Changzhou 213200, China

**Keywords:** deep learning, image dehazing, depth information, bilateral grid

## Abstract

In adverse foggy weather conditions, images captured are adversely affected by natural environmental factors, resulting in reduced image contrast and diminished visibility. Traditional image dehazing methods typically rely on prior knowledge, but their efficacy diminishes in practical, complex environments. Deep learning methods have shown promise in single-image dehazing tasks, but often struggle to fully leverage depth and edge information, leading to blurred edges and incomplete dehazing effects. To address these challenges, this paper proposes a deep-guided bilateral grid feature fusion dehazing network. This network extracts depth information through a dedicated module, derives bilateral grid features via Unet, employs depth information to guide the sampling of bilateral grid features, reconstructs features using a dedicated module, and finally estimates dehazed images through two layers of convolutional layers and residual connections with the original images. The experimental results demonstrate the effectiveness of the proposed method on public datasets, successfully removing fog while preserving image details.

## 1. Introduction

Due to the presence of dust, haze, and other suspended particulates in the atmosphere, visibility is reduced, impacting imaging systems. As light passes through haze and other particulates, refraction and scattering occur [1,2], causing image blur and reduced contrast, which decrease image entropy and affect advanced computer vision tasks, such as object detection and tracking. Image dehazing, as a low-level computer vision task, often serves as a preprocessing step for higher-level vision tasks and thus plays a crucial role in subsequent vision tasks, gradually receiving widespread attention.

Image dehazing aims to recover clean images from hazy counterparts, with foggy images typically described using a physical scattering model.

(1) I(x)=J(x)t(x)+A(1−t(x))

where I(x) is the foggy image to be restored, J(x) is the dehazed image of I(x), t(x) is the transmission function in the air, and A is the global atmospheric light.

(2) $$t(x)=e^{-\beta d(x)}$$

where β represents the scattering coefficient, and d(x) is the distance from the object being photographed to the camera.

To effectively restore foggy images, traditional methods estimate dehazed images through various prior assumptions, such as the dark channel prior [3], color attenuation prior [4], and color lines [5]. However, these prior statistical methods fail to cover all scenarios in real-world scenes, resulting in poor generalization performance.

In recent years, with the rapid development of deep learning technology and its remarkable achievements in the field of computer vision, deep learning-based image dehazing techniques have gradually become mainstream. However, existing image dehazing algorithms have not fully utilized image information and have failed to enhance the edge structure of images, resulting in lower visual quality and blurry edges in dehazed images. Many researchers have begun to incorporate depth information into image dehazing algorithms. For example, Chen et al. utilized the relationship between depth information and transmittance in atmospheric scattering models to propose an end-to-end image dehazing algorithm [6]. This algorithm uses the dark channel prior to provide constraint information to the neural network, generating initial dehazed images, and then refines the dehazed images using depth information. Sharma et al. [7] also utilized depth information and atmospheric values to estimate dehazed images. Compared to other deep learning-based methods, incorporating depth information into image dehazing can leverage the advantages of physical models in improving image visibility. However, such methods still have some drawbacks: (1) although using depth information can improve the overall visual effect of recovered images, the lack of enhancement of image edge information leads to edge blurring in the recovered images; (2) simple addition of image depth information to deep network features can lead to feature reconstruction problems, resulting in the inability to obtain high-quality features.

To address these issues, we propose a deep-guided bilateral grid feature fusion dehazing network. Firstly, an end-to-end training strategy is employed to directly learn the mapping model from hazy images to haze-free images, avoiding color distortion caused by intermediate parameter estimation errors. Secondly, the visual quality of the images is enhanced by the depth information extraction module and bilateral grid construction module, while handling the edge information of the images effectively. The introduction of slice operations on bilateral grids enables feature reconstruction of depth information with bilateral grids. Finally, through two convolutional layers, the generated depth information is fused with bilateral grids to produce clear haze-free images.

The main contributions of this paper are outlined as follows: (1) Introducing a deep-guided bilateral grid feature fusion dehazing network, which employs an end-to-end training strategy to directly learn the mapping model from hazy images to haze-free images. This approach effectively mitigates color distortion caused by intermediate parameter estimation errors. (2) Improving the overall visual quality of images and enhancing edge information by utilizing depth information to guide the sampling of bilateral grid features. This is achieved through the depth information extraction module and bilateral grid construction module. (3) Conducting thorough evaluations on public datasets, demonstrating that the proposed method outperforms seven existing baseline models in terms of image quality, visual effect, and detail preservation in dehazed images.

## 2. Related Work

### 2.1. Single-Image Dehazing Based on Image Enhancement

Image enhancement-based dehazing algorithms typically enhance the contrast of images and correct the colors to improve image quality. This category of algorithms utilizes traditional image processing techniques, such as homomorphic filtering [8], histogram equalization [9,10], and Retinex theory [11,12], to process images. Zhu et al. [13] proposed a novel image dehazing framework based on artificial multi-exposure image fusion, aiming to effectively alleviate the impact of haze on images to achieve high visibility. By performing gamma correction on individual hazy images, a series of artificially under-exposed image sequences are extracted, and brightness and saturation of images are balanced through linear saturation adjustment.

### 2.2. Single-Image Dehazing Based on Prior Knowledge

Methods based on prior knowledge primarily estimate the transmission function t(x) and the global atmospheric light A through various means. Tan [14] based his method on two fundamental experiences: firstly, images affected by severe weather have lower contrast compared to images with high visibility; secondly, atmospheric light mainly depends on the distance between the photographed object and the camera and tends to be smooth. Based on these observations, a dehazing method was proposed using the theory of Markov Random Fields. He [3] and others, by observing outdoor fog-free pictures, derived the dark channel prior theory, and proposed a simple and effective prior-based algorithm for dehazing a single input image.

Berman et al. (2016) [15] argued that the colors of a fog-free image could be approximated by different colors, used color clustering and angles on a spherical coordinate system to identify haze lines, and proposed a non-local prior dehazing method. Zhu [4] et al. innovatively proposed a color attenuation prior, using the color attenuation prior information to restore the depth map of the image, and utilized this depth map for dehazing a single image. Bui and Kim [16] analyzed foggy pixel groups in RGB space to construct color ellipsoids, and based on the geometric shape of these color ellipsoids, calculated transmission values to dehaze the image. Wang et al. [17] proposed a fast single-image dehazing algorithm based on linear transformation. By assuming a linear relationship between the hazy image and the haze-free image in the minimum channel, and accurately estimating the atmospheric light through quad-tree subdivision, it exhibits significant advantages in edge handling and computational speed.

### 2.3. Learning-Based Single Image Dehazing

Early work on image dehazing focused on using neural networks to estimate the transmission function and atmospheric light, and then restore foggy images. Cai [18] and others first proposed a trainable DehazeNet neural network that learns the mapping relationship between foggy images and the transmission function to estimate the transmission function. The estimated transmission function is then used to restore foggy images using the atmospheric scattering model. Ren [19] and colleagues introduced MSCNN, a convolutional neural network for dehazing single images. This model learns from the overall coarse-scale network and local refinement results of blurry images and their corresponding transmission maps to estimate the transmission function, which is then used to dehaze single images. Li [20] and others, through a reconstructed atmospheric scattering model, proposed the lightweight convolutional neural network AOD-Net. This network integrates the transmission map and atmospheric light into a single value, K. By estimating K, a clear image is directly obtained, which can easily be embedded into other vision models. The aforementioned algorithms typically decompose the dehazing problem into two sub-problems: first attempting to estimate the transmission map and global atmospheric light, and then performing image dehazing based on the atmospheric scattering model. However, in the case of a single image, accurately estimating the transmission function and global atmospheric light is often difficult; hence, the dehazing effects are not very satisfactory. Moreover, these methods are not truly end-to-end algorithms and cannot directly map input images to output images. Using deep convolutional neural networks, clear images can be estimated directly from foggy images through stepwise optimization. Ren [21] and others proposed an end-to-end trainable neural network GFN, consisting of an encoder and a decoder. The encoder is used to extract the weight maps and contextual information of the input image, while the decoder estimates the contribution of each input to the final dehazing result based on the feature maps extracted by the encoder, ultimately obtaining a clear image. Xu [22] and others introduced a feature fusion network with an attention mechanism, which can more flexibly handle problems by fusing different types of information. Ping et al. [23] combined high-level semantics and low-level semantics in feature maps of different scales, also increasing attention to different haze concentration areas. Li et al. [24] proposed a network incorporating fog transmission and feature aggregation, which transfers fog information from real foggy images to clear images, generates training samples to address domain shift issues, and improves the scalability of the dehazing model through feature aggregation. Zhang et al. [25] proposed a dual-task collaborative framework that integrates depth estimation and dehazing tasks, achieves single-image dehazing through an interactive mechanism, and optimizes the performance of both tasks through a difference-aware mechanism.

### 2.4. Bilateral Filtering

Barron et al. [26] proposed a bilateral solver that combines the flexibility and speed of simple filtering methods for edge-aware smoothing. Chen et al. [27] introduced bilateral grids to accelerate bilateral filtering, achieving fast edge-aware image processing. Zhang et al. [28] utilized bilateral grids to accelerate depth estimation. Gharbi et al. [29] introduced a neural network architecture inspired by bilateral grid processing and local affine color transformations, learning to make local, global, and content-aware decisions to approximate the desired image transformations. Zhang et al. [30] restored image structure and edge information by fitting multiple affine models in bilateral space.

### 2.5. Monocular Depth Estimation

R. Garg et al. [31] proposed an unsupervised convolutional neural network (CNN) framework for single-view depth prediction, which does not require pre-training or annotated real depth information. Training is performed using a pair of images with known camera motion to predict the depth map of the source image. Clément Godard et al. [32] enhanced performance and robustness by introducing new training losses to ensure consistency between left and right disparity images. Zhou et al. [33] used a single monocular video sequence to train a separate multi-view pose network to estimate the pose between two consecutive frames. Zhou et al. [34] utilized RMSFM to extract per-pixel features, constructed multi-scale feature modulation modules, and iteratively updated inverse depth through a fixed-resolution parameter-sharing decoder to estimate the depth map of a monocular camera.

Currently, methods based on physical models are often limited by the constraints of prior knowledge, which can lead to color distortion in dehazing tasks and poor generalization across different scenes. On the other hand, learning-based methods, while better adapted to various scenes, often do not effectively utilize depth information in the images, resulting in the inability to completely remove fog at greater distances and possible loss of edge information when generating dehazed images. To address these issues, this paper proposes a new dehazing model, including depth estimation and bilateral grid modules. The primary function of the depth estimation module is to extract the image’s depth information, enabling the model to more effectively handle uneven fog situations. The main role of the bilateral grid module is to extract the structural information of object edges, allowing the model to effectively improve edge blurriness. By fusing depth information with bilateral grid information, the model can better handle uneven fog and restore image edge information, thus improving the dehazing effect.

## 3. The Proposed Method

**Figure 1:**
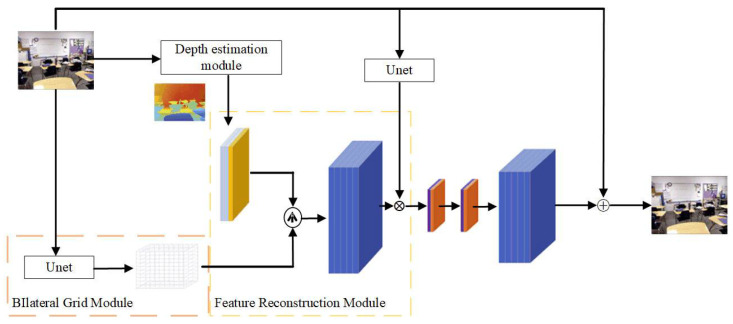
Network architecture.

As illustrated in Figure 1, the original foggy image first passes through the depth estimation module and the bilateral grid module to produce a depth map of the same size as the original and a smaller-sized bilateral grid. Then, guided by the depth map, the bilateral grid undergoes trilinear interpolation sampling to generate a feature map with 12 channels matching the size of the original image. This feature map is then element-wise multiplied and summed with features extracted by a Unet through the feature reconstruction module. Finally, after passing through two convolutional layers, the dehazed image is estimated by making a residual with the original image.

### 3.1. Depth Estimation Module

In this paper, the depth estimation module estimates the depth map [35], where the values of the depth map are related to the distance from the photographed object to the camera.

**Figure 2:**
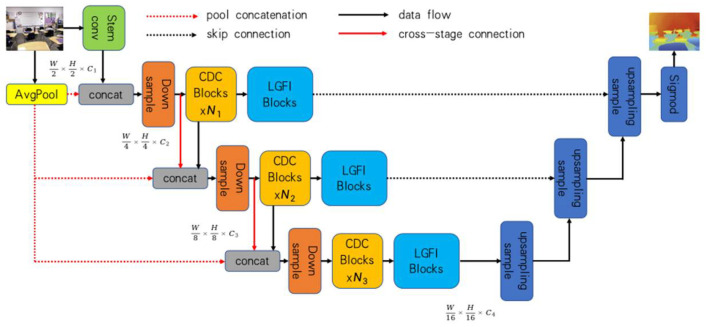
Depth estimation module.

As shown in Figure 2, the depth estimation module consists of an encoder and a decoder. The encoder comprises a series of consecutive dilated convolution modules (CDC) and Local–Global Feature Interaction modules (LGFI). The decoder [36] employs bilinear up-sampling to restore the encoder’s features from four stages into a depth map that matches the input size. The CDC modules utilize dilated convolution to extract multi-scale features. Using different dilation rates at each stage, these modules maintain the size of the output feature map while achieving a larger receptive field. The specific operations of dilated convolution are as follows:

(3) $$y[i]=\sum_{k=1}^{K}x[i+\gamma \times k]w[k]$$

where w represents the values of the convolutional kernel. γ denotes the dilation rate used for the convolutional input x[i].

When the input dimension is *H* × *W* × *C*, the output from the CDC is as follows:

(4) $$\hat{X}=X+L_{PG}(L_{P}(L_{BN}({Conv}_{\gamma }(X))))$$

where $L_{P}$ represents point-wise convolution, and $L_{PG}$ indicates point-wise convolution operation with GELU [37] activation. $L_{BN}\mathrm{represents}\;a\;\mathrm{batch}\;\mathrm{normalization}\;\mathrm{layer},\mathrm{and}{\mathrm{Conv}}_{\gamma }$ is a 3 × 3 dilated convolution with a dilation rate of *γ*.

When the LGFI module receives an input feature map of dimensions *H* × *W* × *C* denoted as X, the feature map is first mapped to *Q*, *K*, and *V* with the specific expressions as follows:

(5) $$Q=XW_{q},K=XW_{k},V=XW_{v}$$

(6) $$\tilde{X}=Attention(Q,K,V)+X$$

(7) $$\hat{X}=\tilde{X}+L_{PG}(L_{P}(L_{LN}(\tilde{X})))$$

where $W_{q}$, $W_{k}$, $W_{v}$ are learnable parameters, and Attention(Q,K,V) is the cross-covariance attention [38] mechanism:

(8) $$Attention(Q,K,V)=V\cdot Softmax(Q^{T}\cdot K)$$

### 3.2. Bilateral Grid Module

**Figure 3:**
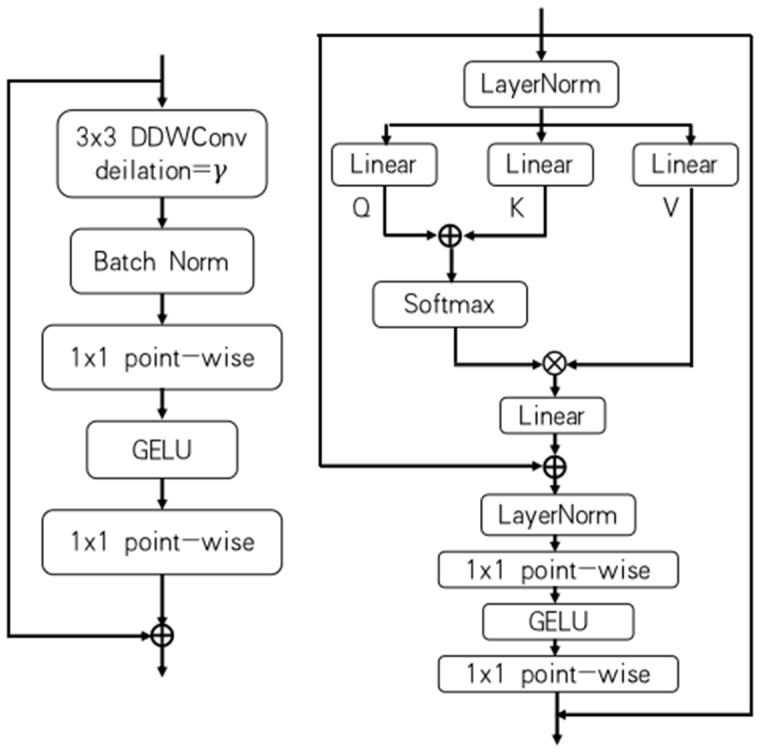
CDC module (**left**) LGFI module (**right**).

Due to factors such as low contrast and blurred edges in foggy images, the intensity values of two pixels at a boundary can be very similar. Using bilateral filtering, the edge information of the image can be better processed. As shown in Figure 3, the image is first resized to (256, 256) using bicubic interpolation, and multi-scale features of the image are extracted through a Unet [39], generating a feature map the same size as the original image. This feature map is then converted into a low-resolution bilateral grid.

### 3.3. Feature Reconstruction Module

**Figure 4:**
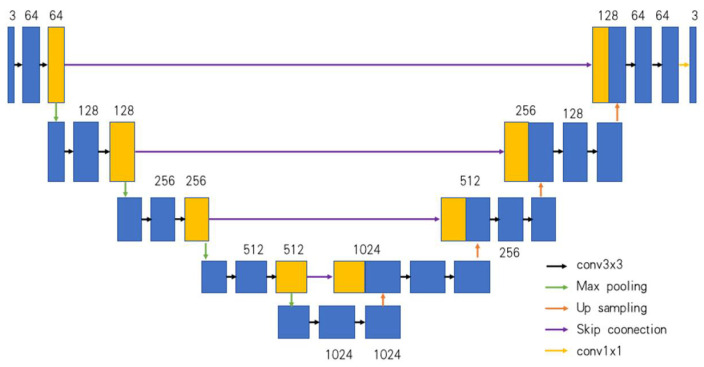
Unet neural network.

**Figure 5:**
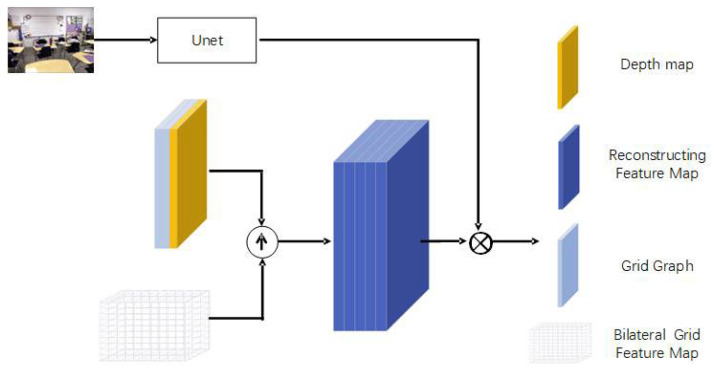
Feature reconstruction module.

To better utilize the low-resolution features extracted from the bilateral grid, this paper proposes a bilateral grid slicing method guided by depth information. As shown in Figure 4, the depth map is first up-sampled to the original image size using bilinear interpolation, and then the up-sampled depth map is concatenated with two grid maps to form a three-channel sampling map. This three-channel sampling map is then used to sample the bilateral grid using trilinear interpolation to form a feature map of the same width and height as the original image, with 12 channels reconstructed feature map. Finally, an element-wise dot product is performed with the features extracted by Unet from the feature reconstruction module shown in Figure 5, producing a reconstructed feature map with three channels, the same width and height as the original image. The specific operations are as follows:

(9) $${\hat{C}}_{i}=\sum_{k=4*i}^{4*i+3}C_{i}*f(k)+f(4*i+4),i=1,2,3$$

where $C_{i}$ is the feature of the *i*-th channel extracted by Unet. f(k) is the sampled feature map, and ${\hat{C}}_{i}$ is the output of the feature reconstruction module.

### 3.4. Loss Functions

The network model in this paper employs loss functions that include the L1 loss function and the perceptual loss function. The perceptual loss function uses the MSE of the feature maps output by the first convolutional layer of the third block of VGG19, which has a receptive field of 68 × 68. It draws from the discriminator of the generative adversarial network’s PatchGan [40], enabling the model to better learn the details, style, and color of the images.

(10) $$L={\lambda_{1}L}_{1}+\lambda_{2}L_{p}$$

The L1 loss function is specified as follows:

(11) $$L_{1}=\frac{1}{N}\sum_{i=1}^{N}|I(i)-I_{haze}(i){|}_{1}$$

where I(i) represents the value of the pixel at the *i*-th position of the clear image, and $I_{haze}$ represents the value of the pixel at the *i*-th position of the foggy image.

The Lp loss function is specified as follows:

(12) $$L_{p}=\frac{1}{H\times W\times C}\sum {|VGG}_{N}(I)-{VGG}_{N}(I_{haze}){|}_{2}$$

where ${VGG}_{N}$ represents the feature map of VGG19 with a receptive field of *N* × *N*, **I** represents the clear image, and $I_{haze}$ represents the foggy image.

## 4. Dataset and Experimental Setup

The dataset used in this paper is sourced from the RESIDE dataset [41], which is widely used in image dehazing tasks. It primarily consists of five subsets: indoor training set (ITS), outdoor training set (OTS), synthetic objective testing set (SOTS), hybrid subjective testing set (HSTS), and real world task-driven testing set (RTTS). In this experiment, the network is trained using the indoor training set (ITS) and outdoor training set (OTS). To verify the effectiveness of the proposed algorithm, the synthetic objective testing set (SOTS) and hybrid subjective testing set (HSTS) are used for testing.

The image dehazing network proposed in this paper is implemented in Python, using the PyTorch 1.9.0 framework on an Ubuntu 20.04 operating system, with a 3.50 GHz Intel Core i9-11900K CPU (Intel Corporation, located in Santa Clara, CA, USA) and an NVIDIA 3090 GPU (NVIDIA Corporation, located in Santa Clara, CA, USA), accelerated by CUDA 11.0. The batch training size is set to 8, and the total number of training epochs is set to 50. The model uses the Adam optimizer for optimization, with an initial learning rate set at 0.0001, the first momentum term set at 0.9, and the second momentum term set at 0.9999. The learning rate is adjusted to 10 percent of its original value at epochs 10, 20, and 40, with $\lambda_{1}$ = 0.7 and $\lambda_{2}\;$= 0.3.

## 5. Evaluation Metrics

The evaluation metrics used in this paper are PSNR and SSIM. PSNR is widely used in the objective evaluation of image restoration tasks to measure the quality of processed images. Given two images, PSNR calculates the pixel-by-pixel difference between them; a higher PSNR indicates smaller differences between the two images, signifying better image quality. SSIM is used to measure the similarity between two images, evaluating them in terms of brightness, contrast, and structure. The closer the SSIM value is to 1, the more similar the two images are.

### 5.1. Experimental Results and Evaluation

#### 5.1.1. Quantitative Analysis

The testing dataset used in this paper consists of 500 indoor and 500 outdoor images from SOTS, and 10 outdoor images from HSTS. The comparison algorithms used include DCP [3], AOD-NET [20], HRN [42], EPDN [43], RefineDnet [44], HDDNet [45], and SDNet [46] with all comparisons conducted under the same experimental conditions.

As shown in Table 1, on the SOTS indoor paired test set, the PSNR performance metric of the proposed algorithm is 26.99 dB, which is an improvement of 10.37 dB, 7.93 dB, 5.97 dB, 1.93 dB, 2.76 dB, 2.2 dB, and 2.08 dB over the DCP, AODnet, HRN, EPDN, RefineDnet, HDDNet, and SDNet, respectively; the SSIM performance metric is 0.9343, which is an improvement of 0.1164, 0.0839, 0.0665, and 0.0437 over the DCP, AODnet, HRN, and EPDN, respectively.

As seen in Table 2, on the SOTS outdoor test set, the PSNR performance metric of this paper’s algorithm is 27.69 dB, which represents an improvement of 8.56 dB, 7.40 dB, 1.28 dB, 5.12 dB, 6.64 dB, 5.17 dB, and 3.13 dB over the DCP, AODNet, HRN, EPDN, RefineDnet, HDDNet, and SDNet, respectively; in terms of SSIM performance metric, it is 0.9156, an improvement of 0.1008, 0.0391, 0.0027, 0.0526, 0.0039, 0.0056, and 0.0056 compared to the DCP, AODNet, HRN, EPDN, RefineDnet, HDDNet, and SDNet algorithms, respectively. The metrics of this paper’s algorithm are higher than those of the comparison algorithms.

As detailed in Table 3, on the HSTS test set, the PSNR performance metric of this paper’s algorithm is 29.52 dB, an improvement of 12.73 dB, 9.93 dB, 3.20 dB, 8.00 dB, and 8.33 dB over the DCP, AODNet, HRN, EPDN, and RefineDnet, respectively; in terms of SSIM performance metric, it is 0.9136, an improvement of 0.0560, 0.0726, 0.0279, 0.0285, and 0.0211 compared to the DCP, AODNet, HRN, EPDN, and RefineDnet, respectively.

#### 5.1.2. Qualitative Analysis

**Figure 6:**
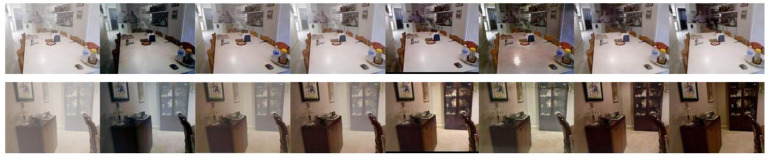
Comparison chart of indoor dehazing methods using SOTS. From left to right are, respectively, original image, DCP, AODNet HRN, EPDN, RefinedDnet, and our algorithm.

As shown in Figure 6, the DCP, AODNet, HRN, EPDN, and RefinedDnet algorithms did not completely remove the fog from the images. The DCP, AODNet, HRN, and RefinedDnet algorithms, compared to the EPDN and our algorithm, did not handle distant fog effectively, leaving a significant amount of fog. The dehazed images estimated by the RefinedDnet and DCP algorithms have lower brightness and some color distortion. The RefinedDnet and AODNet algorithms resulted in some over-saturation of colors. Our proposed algorithm provided the best dehazing effect, closest to the original clear image.

**Figure 7:**
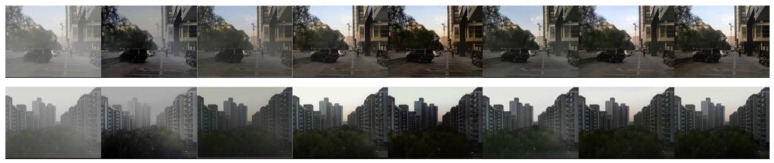
Comparison chart of outdoor dehazing methods using SOTS. From left to right are, respectively, original image, DCP, AODNet, HRN, EPDN, RefinedDnet, and our algorithm.

As depicted in Figure 7, our algorithm, by integrating depth information, handled distant fog more cleanly compared to other algorithms. The DCP, AODNet, HRN, EPDN, and RefinedDnet algorithms did not completely remove the fog. The DCP, AODNet, and RefinedDnet algorithms performed poorly on distant fog, and the dehazed images estimated by the EPDN algorithm had lower brightness. Our proposed algorithm offered the best dehazing effect, closest to the original clear image.

**Figure 8:**
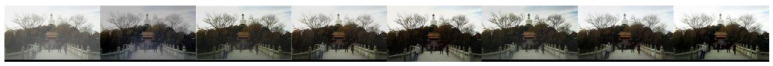
Comparison chart of outdoor dehazing methods using HSTS. From left to right are, respectively, original image, DCP, AODNet, HRN, EPDN, RefinedDnet, and our algorithm.

As depicted in Figure 8, the images processed by the DCP, AODNet, HRN, and RefinedDnet algorithms were able to remove the fog, but not completely, leaving a thin layer of haze. The images processed by the EPDN algorithm appeared dimmer than the original clear image. Except for the algorithm proposed in this paper, the other methods did not handle the sky regions well, resulting in some dark areas.

**Figure 9:**
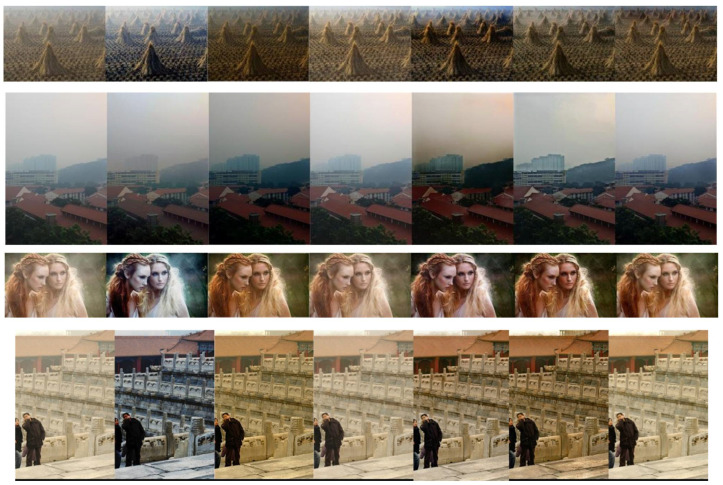
Comparison chart of dehazing effectiveness for real foggy weather conditions. From left to right are, respectively, original image, DCP, AODNet, HRN, EPDN, RefinedDnet, and our algorithm.

As shown in Figure 9, in terms of real foggy dehazing effects, the image processed by the DCP algorithm overall appears darker and slightly distorted in color. The AODnet and EPDN algorithms suffer from low brightness issues, particularly in handling distant fog, which can lead to image distortion. The RefinedDnet algorithm results in overly saturated colors and blurring at the edges in the second image. Compared to these methods, the algorithm proposed in this paper effectively dehazes while maintaining good visual quality, avoiding the aforementioned issues.

Compared with several existing image dehazing models, AODNet and EPDN exhibit lower FLOPS and parameter counts, indicating a lower model complexity. This may reduce performance when dealing with challenging dehazing details. In contrast, HRN and RefineDnet have higher FLOPS, which might lead to insufficient performance in environments with limited computational resources, as shown in Table 4. Our model achieves a good balance between dehazing effect and computational complexity, ensuring excellent dehazing performance while maintaining high computational efficiency.

### 5.2. Ablation Study

Ablation Study of Network Architecture: In this study, we only used the L1 loss and removed the depth estimation module, bi-lateral grid module, and feature reconstruction module to obtain the baseline model (a). Additionally, we removed the depth estimation module (b). Loss Function Ablation Study: We used the complete network structure and conducted experiments with only the L1 loss (c), L1 loss combined with perceptual loss using a 5 × 5 receptive field extracted from VGG19 (d), and L1 loss combined with perceptual loss using a 252 × 252 receptive field extracted from VGG19 (e). The proposed method (f). The ablation experiments were conducted on the RESIDE SOTS indoor testing set.

The loss function used in this algorithm is:

(13) $$L={\lambda_{1}L}_{1}+\lambda_{2}L_{p}$$

where $L_{1}$ calculates the pixel-wise difference, primarily extracting the low-frequency information of the original image, and $L_{p}$ perceptual loss is utilized to extract the high-level features of the image, including color, texture, high-frequency, and style information.

As shown in Table 5, the baseline model achieves a PSNR of 24.82 dB and an SSIM of 0.9128. Adding bilateral grid information improves the network performance, with a PSNR increase of 0.11, but the SSIM does not increase, indicating that using bilateral grid information alone does not significantly improve the network. After incorporating both depth information and bilateral grid information, the network’s performance improves significantly, with PSNR increasing to 25.21 dB and SSIM increasing to 0.9202. This indicates that introducing both depth information and bilateral grid information contributes to the network’s performance. When only using a receptive field of 68 × 68, the model’s performance drastically decreases due to the lack of low-frequency information, with PSNR only at 20.69 dB and SSIM at 0.867.

Using only the *L*_1_ loss function, the PSNR on the SOTS indoor dataset is 25.21 dB, and the SSIM is 0.9202. The PSNR of our algorithm using only the *L*_1_ loss function still exceeds the performance levels of the DCP, AODNet, EPDN, RefineDnet, and HRN algorithms.

Further testing of three types of perceptual losses was conducted, specifically for receptive fields of 5 × 5, 68 × 68, and 252 × 252 feature layers’ mean squared error (MSE). Using the MSE output of a receptive field of 5 × 5 as the loss function, performance met-rics improved by 0.82 dB. Similarly, using the MSE output of a receptive field of 252 × 252 feature layer, the PSNR increased by 0.81 dB, and SSIM improved by 0.0043.

Since the performance improvements between the MSE outputs of receptive fields of 5 × 5 and 252 × 252 feature layers were minimal, inspired by the PatchGan discriminator, a new approach was attempted. We used the MSE of VGG19 with a receptive field of 68 × 68 as the perceptual loss, to learn the high-frequency components, color, and style information of the image. The results showed significant improvements in both PSNR and SSIM, increasing by 1.78 dB and 0.0141, respectively. On the SOTS indoor dataset, when the loss function utilized the perceptual loss output of the feature map with a receptive field of 68 × 68, the model performance reached its optimal level.

## 6. Conclusions

Methods based on physical models are often constrained by prior knowledge, which may lead to color distortions in the dehazed images and poor generalizability across different scenes. Additionally, while learning-based methods offer better adaptability, they often fail to fully utilize the depth information in images. This results in poor performance in handling distant fog and restoring image edges.

To overcome these issues, this paper proposes a novel dehazing model composed of a depth estimation module and a bilateral network module. The depth estimation module extracts depth information from the input image, enabling the model to more effectively handle uneven fog situations. Through a strategy of depth-guided bilateral grids, the depth information is integrated with bilateral grid data, allowing the network to better restore the edge information of images, thereby significantly improving the dehazing effect.

## 7. Future Work

We plan to deploy the proposed image dehazing network based on depth information and bilateral grids onto the NVIDIA Jetson Nano development board to achieve real-time image dehazing inference. We will adopt lighter modules and optimize the model for the hardware architecture of NVIDIA Jetson Nano, reducing model parameters and computational complexity. We will develop real-time image processing applications, capture input images using a camera, perform real-time inference, and optimize data flow to improve processing speed. Testing will be conducted in real-world application scenarios to verify the system’s stability and dehazing effect, providing strong support for practical applications.

## Figures and Tables

**Table 1:** Comparison of various algorithms on RESIDE SOTS indoor testing set.

	DCP	AODNet	HRN	EPDN	RefineDnet	HDDNet	SDNet	Ours
PSNR	16.62	19.06	21.02	25.06	24.23	24.79	24.91	26.99
SSIM	0.8179	0.8504	0.8678	0.8906	0.9431	0.9400	0.9400	0.9343

**Table 2:** Comparison of various algorithms on RESIDE SOTS outdoor testing set.

	DCP	AODNet	HRN	EPDN	RefineDnet	HDDNet	SDNet	Ours
PSNR	19.13	20.29	26.41	22.57	21.05	22.52	24.56	27.69
SSIM	0.8148	0.8765	0.9129	0.8630	0.9117	0.9100	0.9100	0.9156

**Table 3:** Comparison of various algorithms on RESIDE HSTS testing set.

	DCP	AODNet	HRN	EPDN	RefineDnet	Ours
PSNR	16.79	19.59	26.32	21.52	21.19	29.52
SSIM	0.8576	0.8410	0.8857	0.8851	0.8925	0.9136

**Table 4:** Comparison of FLOPS and parameter count for various image dehazing models.

	DCP	AODNet	HRN	EPDN	RefineDnet	HDDNet	SDNet	Ours
FLOPS	--	512.5 M	125.9 G	22.71 G	258.43 G	--	--	107.58 G
parameters	--	1.76 k	2.26 M	17.38 M	11.38 M	0.458 M	--	39.47 M

**Table 5:** Comparative ablation experiments on the RESIDE SOTS indoor testing set.

Loss Functions	PSNR	SSIM
a	24.82	0.9128
b	24.93	0.9016
c	25.21	0.9202
d	26.03	0.9201
e	26.02	0.9245
f	26.99	0.9343

## Data Availability

The data presented in this study are available on request from the corresponding author.
